# Editorial: A review of canine soft tissue sarcomas: new insights in diagnostic and treatment measures

**DOI:** 10.3389/fvets.2024.1454513

**Published:** 2024-07-10

**Authors:** Giulia Moretti, Antonello Bufalari

**Affiliations:** Department of Veterinary Medicine, University of Perugia, Perugia, Italy

**Keywords:** soft tissue sarcoma, canine, advances, electrochemotherapy, microbrachytherapy

Soft tissue sarcoma (STS) is a group of mesenchymal tumors which differ each other but have similar histological appearance and clinical-biological behavior (1–4). They account for 8–15 and 7–18% of all cutaneous and subcutaneous tumors in dogs and cats, respectively. In the dog, STS preferentially develop in medium and large sized dogs, ranging in age from 5 to 17 years. The group of STS includes fibrosarcoma, liposarcoma, leiomyosarcoma, perivascular tumors, rhabdomyosarcoma, malignant fibrous histiocytoma, myxosarcoma, mesenchymoma, tumors of peripheral nerve sheaths, not originating from plexuses, and undifferentiated sarcomas. Histologically they can be classified as grade I (low), II (intermediate), and III (high) (5). Grade I STS prevails (up to 84%) while grade III STS is rare (up to 7%) (6, 7). The proportion of high-grade STS increases to 29% in studies derived from referral clinics (8, 9). Excluded from the group of STS are hemangiosarcoma, synovial sarcoma, gastro-intestinal stromal tumor, oral fibrosarcoma, and peripheral nerve sheath tumors localized to the brachial plexus or lumbosacral plexus (10). The main features of STS are development in any part of the body (although more than 50% develop in the limbs, 35% in the trunk, and 5% in the skull), being surrounded by pseudocapsules, tendency to infiltrate fascial planes, high recurrence rate after conservative surgery, metastasis by haematogenous route and poor response to both chemo- and radiotherapy if macroscopic lesion is still present (5, 10). The known etiology of STS is related to isolated cases: previous trauma, parasites (*Spirocerca lupi*), and implants (11–14). Surgery is the treatment of choice, but depending on histotype, histological grade, margin status, and clinical stage, other oncological therapies should sometimes be considered, including radiotherapy (RT), electrochemotherapy (ECT), traditional chemotherapy, intralesional chemotherapy, and metronomic therapy. Electroporation-based treatments have been proven to be safe and effective in veterinary oncology, although they have not been accepted as standard treatments, especially in oral and maxillofacial oncology (15). As previously reported in the literature, ECT is a treatment recommended mostly in cases in which the owners decline surgery and/or radiotherapy (16). Unfortunately, in veterinary medicine, few studies have evaluated the role of these adjuvant therapies, with low scientific evidence, mostly due to the retrospective nature of the studies, the small sample size, and the lack of a control population (17).

This Research Topic comprises five original research publications from a number of both clinical and pathologist researchers. Taken together, these articles are geared toward the advancement of our understanding, diagnosis, and treatment of sarcomas in dogs by researching new therapeutic approach and combinations. Recent technologies are used in the original research articles with particular concerns about ECT.

Brloznik et al. investigate the correlation of dynamic contrast-enhanced ultrasound (DCE-US) results with therapy outcomes in 12 canine soft tissue tumors (11 mast cell tumors and one neurofibrosarcoma) treated with ECT combined with encoding interleukin-12 (GET pIL-12). ECT and/or gene electrotransfer of plasmid DNA GET pIL-12 are effective treatments for canine cutaneous, subcutaneous and maxillofacial tumors. They performed clinical follow-up examinations using DCE-US with short and long term follow-up after treatment. Perfusion heterogeneity is noted as a hallmark of malignant tumors and also provides valuable information for discriminating malignant from benign lesions. Numerous significant differences in DCE-US parameters were noted from the authors between tumors with completer remission (CR) and non-CR tumors showing that perfusion and perfusion heterogeneity were lower in CR tumors than in non-CR tumors. These important findings indicates a possible predictive value of perfusion heterogeneity in antitumor therapies based on anti-angiogenic effects.

Morsink et al. evaluate the safety and efficacy of ^166^Ho microbrachytherapy in seven client-owned canine patients with soft tissue sarcomas. The results of this study show that ^166^Ho microbrachytherapy was a safe and effective neoadjuvant treatment option for canine patients with STS with limited and resolved side effects: one case presented local necrosis and another one ulceration of the skin covering the tumor. ^166^Ho is a promising radionuclide for micro-brachytherapy because it emits high energy beta radiation with a short soft tissue penetration depth, thereby enabling a high tumor dose with minimal risk for surrounding tissues; thus, this could be considered a new, minimally invasive neoadjuvant treatment option for inoperable solid malignancies. The authors obtained a tumor volume reduction of 49.0 ± 21.3% which facilitated marginal surgical resection of residual tumor and attributed to long survival times, also for relatively large tumors.

The third paper by Moretti et al. was a retrospective and descriptive study including 19 dogs with various tumor type of the oral cavity treated with ECT coupled with bleomycin administration. Several clinical and ECT electrical parameters has been collected and analyzed with the aim to compared the ECT efficacy between different histological subtypes. Ideal anticancer therapies should be highly efficacious, widely available at low cost, and associated with a minor risk of causing adverse events: repeated ECT application coupled with bleomycin, resulted safe and effective especially in local tumor control and should be considered as a valid therapeutic option. Despite the heterogeneity of the tumor types included their study, the results confirm that electroporation-based treatments are safe, simple, fast and effective alternatives for selected oral tumors, but there is currently no consensus on timing and the quantity of retreatments so further studies are needed to standardize ECT protocols.

The last paper of this Research Topic by Leonardi et al., aims to identify new and interesting biomolecular and immunohistochemical aspects of canine extraskeletal osteosarcoma (EOS), even though it does not primarily focus on soft tissue sarcomas (STS). This study particularly focuses on the expression of two main markers: RUNX2 and karyopherin alpha-2. The purpose of this paper is to provide a comparative analysis between EOS and primary osteosarcomas of the bone (POS). The most significant morphological and immunohistochemical differences were found to be correlated with the degree of bone matrix formation and the characteristics of tissue diffusion and invasion. Immunohistochemistry can sometimes prove helpful in defining the diagnosis through markers, and the results of this study suggest that karyopherin alpha-2 and RUNX2 could serve as additional diagnostic tools to improve the specificity and sensitivity of osteosarcoma diagnoses, even in extraskeletal cases. The study's data relating to the RUNX2 immunoreactivity of osteoblasts in the areas adjacent to the osteoid deposits in POS, strongly encourage a more sophisticated pursuit of authors' investigative work, exploring deeper in all the different forms of osteosarcoma.

Despite all the existing literature and evidence related to this extremely important Research Topic, the papers published in this Research Topic clearly show that there are still many aspects to be clarified and understood in veterinary oncology. Authors sincerely hope that readers will enjoy reading these original contributions that remind us also of the crucial importance of interdisciplinary collaboration between those working on oncology patients in veterinary medicine. Future progress will be significantly enhanced if these figures communicated and collaborated more effectively.

## Author contributions

GM: Conceptualization, Writing – original draft, Writing – review & editing. AB: Conceptualization, Supervision, Writing – original draft, Writing – review & editing.
